# Effects of maize plant type and row width in ginger/maize intercropping system on yield, economy, and interspecific competition

**DOI:** 10.3389/fpls.2026.1798681

**Published:** 2026-05-29

**Authors:** Ailin Tian, Linyu Liu, Haodan Zhang, Rui He, Junlan Liu, Guoqing Sun, Qin Huang, Sida Long, Bingxia Yang, Xu Yan, Meixia Zhang, Yun Ren, Qiang Li

**Affiliations:** 1 Chongqing Key Laboratory for Germplasm Innovation for Special Aromatic Spice Plants, College of Smart Agriculture/Institute of Special Plants, Chongqing University of Arts and Sciences, Chongqing, China; 2 Chengdu Academy of Agriculture and Forestry Sciences, Chengdu, Sichuan, China

**Keywords:** economy, ginger, intercropping, maize, yield

## Abstract

Ginger/maize intercropping system improves land use efficiency and sustainable agriculture. Field experiments (with sole-cropped ginger (SG) and sole-cropped maize (SM) as controls) tested two maize plant types (flat and compact) and three row widths (2, 3, 4 m). Compared to sole cropping, intercropping reduced maize yield but increased ginger yield and total yield due to ginger’s competitive advantage, yielding higher economic benefits. Land equivalent ratio (LER) and system productivity index (SPI) reached 2.07 and 67.46, respectively, and the benefit-cost ratio (BCR) of the ginger/maize intercropping system was 1.28 to 1.65 times that of sole-cropped maize, showing obvious economic advantages. The aggressiveness (A) and competitive ratio (CR) of ginger were significantly higher than those of compact maize (ZH 2), while maize was the opposite. Therefore, all benefit indicators (LER, SPI, actual yield loss (AYL)), intercropping advantage (IA), monetary advantage index (MAI) and BCR were significantly higher in the flat maize treatment than in the compact maize treatment. Increasing row width significantly reduced the A and CR of ginger, but increased the A and CR of maize, resulting in a significant reduction in ginger, maize and total yield, and a significant reduction in economic benefits. In conclusion, in the ginger/maize intercropping system, the intercropping of ginger and flat maize under 2 m row spacing produced the weakest interspecific competition. Ginger had a competitive advantage and maize was at a competitive disadvantage. Under this configuration, the yields of ginger, maize, and the total system yield all reach the optimal level, with the most significant economic benefits.

## Graphical Abstract

**Figure:**
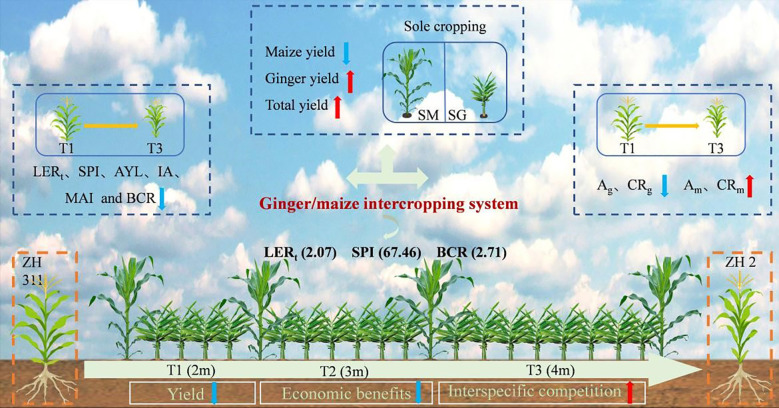

## 1 Introduction

Ginger (*Zingiber officinale* Roscoe), a perennial herbaceous plant of the Zingiberaceae family, is rich in proteins and minerals and contains active compounds such as gingerols and flavonoids. Due to its unique aromatic odor, it is widely used in dietary applications, pharmacy, and perfumery (Guo et al., 2020; Xiao et al., 2024; Gao and Di, 2022). Ginger originates in tropical and subtropical regions of Asia and is currently cultivated in > 40 countries worldwide (Hu, 2023; Ji et al., 2023). China is the world’s leading producer of ginger, with an annual planting area of > 250,000 hectares, exceeding 50% of the total global ginger cultivation area. Its total annual output exceeds 8 million tons, accounting for approximately 2/3 of the world’s ginger production. China is also the largest ginger exporting country, with its export volume accounting for approximately 3/4 of the total global ginger trade volume. Therefore, the sustainable and robust development of China’s ginger industry will substantially impact the global ginger trade (Zu et al., 2024).

Ginger prefers cool, humid environments. Its root system is shallowly distributed, endowing it with poor drought resistance and rendering it intolerant to high temperatures and intense sunlight (Yu et al., 2021). Under the conditions of high temperature and intense sunlight in summer, ginger leaves accumulate large amounts of harmful substances such as Malondialdehyde (MDA), OH^-^, and O_2_^-^. This leads to a reduction in photosynthetic efficiency, poor growth and development of ginger, and a decline in yield (Lyu, 2022; Wang et al., 2024). In contrast, under shade conditions, the chlorophyll content in ginger leaves increases, the photosynthetic photon flux density is decreased, leaf temperatures drop, stomatal conductance and intercellular CO_2_ concentrations decline, and the photosynthetic rate is increased, which promotes the growth and development of ginger and increases its yield (Shafiq et al., 2020; Zhang and Xu, 2008; Zhu Y. et al., 2025). Therefore, shading during summer is of the utmost importance for ginger production.

Traditional artificial sheds increase production costs and labor intensity. Consequently, in recent years, the shading method of ginger has gradually transitioned from artificial shed to intercropping with tall crops (Guan et al., 2007; Peng et al., 2023; Tan et al., 2013). Intercropping, which is characterized by a significant overlapping growth period of two or more crop species on the same field within a growing season, enables complementary utilization of aerial resources (e.g., light and temperature) and belowground resources (e.g., nutrients and water) through strategic crop combinations. This facilitation allows for a more efficient use of agricultural resources, provides a natural shading environment for shade-tolerant crops like ginger, and ultimately enhances crop yield. In the ginger/sweet corn intercropping system, the highest ginger yield was obtained at the planting ratio of 2: 2, and the economic benefit of the intercropping system reached the maximum (Sharma et al., 2023). intercropping system of passion fruit/ginger not only benefits the growth of passion fruit, but also gives full play to the geographical advantages of passion fruit in providing shade and heat dissipation. This creates a favorable growth environment for ginger, achieving a double yield increase for both passion fruit and ginger (Su, 2015). In the study of the effect of maize plant type characteristics on population structure, it was found that maize plant type directly determines the microclimate and light environment of the lower layer crops by regulating the way of sunlight penetrating the canopy (Feng, 2020). In our previous study, we found that under ginger/maize intercropping system, growth performance of ginger improved effectively, leading to a substantial increase in yield. Therefore, ginger/maize intercropping system maximizes the use of light, reduces production costs, and enhances economic benefits, demonstrating broad promotion value.

Numerous studies have been published concerning the effects of intercropping system on crop photosynthetic performance, growth characteristics, yield, and nutrient utilization. Nevertheless, previous studies mainly focused on sun-loving understory crops such as potato, maize (Huang et al., 2013), soybean (Fan et al., 2016) and sweet potato (Xiao et al., 2013). By comparison, relatively few studies have been carried out on yield performance, interspecific competition and economic benefits of the intercropping system of shade-tolerant ginger with maize. In this intercropping system, compared with monoculture, the combination of specific row width and maize plant type may optimize interspecific resource allocation, reduce interspecific competition intensity, improve the photosynthetic performance and nutrient utilization efficiency of ginger and maize, thereby increasing crop total yield and economic benefits.It is worth noting that intercropping is an intrinsically complex system whose performance is co-determined by crop allocation, resource supply, environmental conditions and interspecific interactions (Franca et al., 2025). In accordance, such complexity has been widely reported in multiple cropping research, and comprehensive evaluation with scientific approaches and multi-index systems is essential to reveal the internal mechanisms and comprehensive benefits of intercropping systems (Balandaite et al., 2024). In the study of maize intercropping soybean, it was found that soybean intercropping compact maize could obtain higher yield, while intercropping loose maize could increase soybean shade and reduce yield (Wang et al., 2008). When exploring the effects of different row ratio configurations on intercropping soybean, it was found that when the row ratio of maize and soybean was 4: 4 and the bandwidth was 4.4 meters, the highest population yield and economic benefits were obtained (Zhu G. X. et al., 2025). Therefore, this study selected two maize plant types under three row width treatments to investigate the effects of ginger/maize intercropping system on crop yields, economic benefits, and interspecific competition, and to provide a theoretical basis and technical support for the exploration of ginger/maize intercropping system and reasonable row width selection.

## 2 Materials and methods

### 2.1 Experiment material

The experimental materials included two maize varieties with different plant types, ‘*Zhenghong* 311’ (ZH 311) and ‘*Zhenghong* 2’ (ZH 2), as well as ‘*Yujiang* 1,’ the main ginger variety cultivated in the southwestern region of China. Characteristics of the maize varieties are listed in **Table 1**.

**Table 1:** Experimental crop variety characteristics.

Cultivar	Plant type	Plant height/cm	Yield t/ha	Growth period/d
ZH 311	flat	290	8.22	115
ZH 2	compact	262	7.65	124

### 2.2 Research location

**Figure 1:**
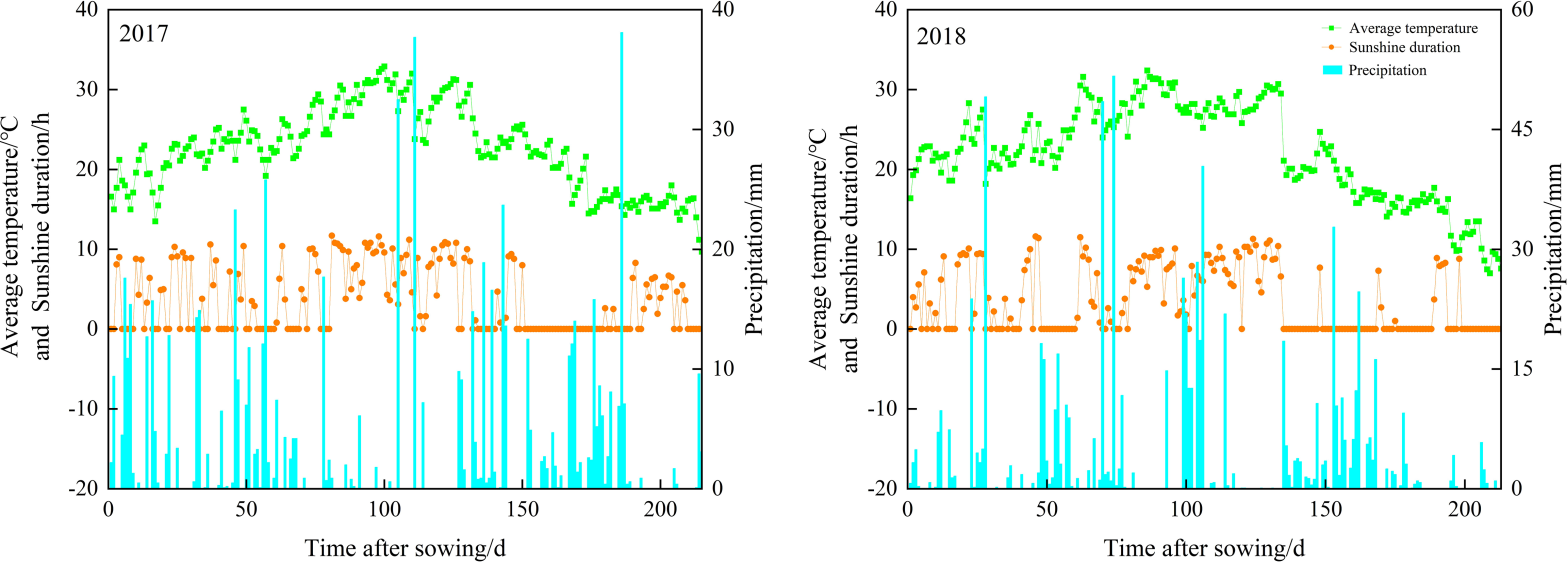
Meteorological conditions during ginger growth period. Including the average temperature (°C), sunshine hours (h) and precipitation (mm).

The experiment was conducted from 2017 to 2018 at a ginger planting base in Wujian Town, Yongchuan District, Chongqing University of Arts and Sciences, which is part of the subtropical monsoonal humid climate zone. Soil in the experimental field, the meteorological conditions during the growth period of ginger are shown in **Figure 1**. within a depth of 0–30 cm, contained 40.8 mg kg^-1^ of alkaline hydrolyzable nitrogen, 34.2 mg kg^-1^ of available phosphorus, 297.6 mg kg^-1^ of available potassium, 1.2 g kg^-1^ of total nitrogen, 0.4 g kg^-1^ of total phosphorus, 1.7 g kg^-1^ of total potassium, and 18.3 g kg^-1^ of organic matter, with a pH value of 6.73.

### 2.3 Experimental design

**Figure 2:**
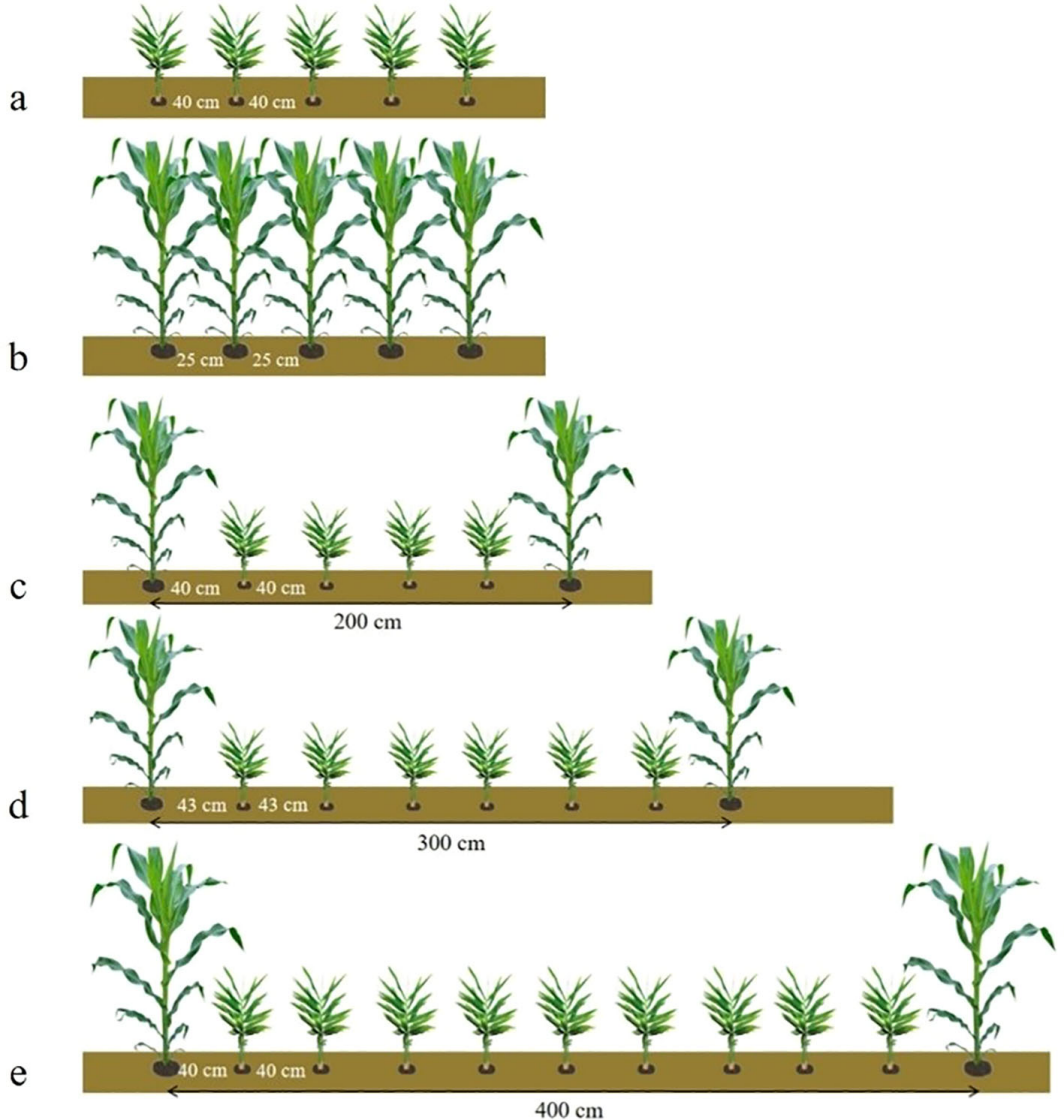
Schematic diagram of ginger/maize intercropping system in the field. In the three planting patterns utilized in this research, **(a, b)** represent the sole-cropped ginger (SG) and sole-cropped maize (SM), different row widths are set for distances of 2 m **(c)**, 3 m **(d)** and 4 m **(e)**.

The experiment was conducted in a 48 m^2^ (12 × 4 m) plot with a randomized block design. There were 8 treatments with 3 replicates per treatment. Two types of maize varieties (flat-type maize ZH 311 and compact-type maize ZH 2) were set up and treated in three row-width configurations (**Figure 2**). Row widths for ginger and maize cultivation were set to 2 m (T1), 3 m (T2), and 4 m (T3). The two control treatments (CK) were sole-cropped ginger without shading (SG) and sole-cropped maize without shading (SM). Ginger adopted a direct seeding method; the planting direction was north-south, and the plant spacing was 0.4 m. To ensure the uniformity of maize seedlings and the coordination with ginger planting configuration, maize seedlings were transplanted after being raised. The transplantation was conducted in a single-plant and single-row arrangement, following the same planting direction as ginger, with a plant spacing of 0.25 m. The planting density of crops in ginger/maize intercropping system is shown in **Table 2**. Maize was transplanted on May 10, 2017, and harvested on August 30, 2017, while in 2018, maize was transplanted on May 8 and harvested on August 27. Ginger was sown on April 20, 2017, harvested on November 20, 2017, and in 2018, it was sown on April 25 and harvested on November 23. The mature period of maize is 120 days, and the mature period of ginger is 210 days. During the test, maize matured earlier and was harvested preferentially. Before sowing, the experimental field was evenly plowed, and all crops were uniformly fertilized. Phosphorus-potassium fertilizer and organic fertilizer were applied as base fertilizers simultaneously, and nitrogen fertilizer was applied twice in equal proportions (50% as base fertilizer and 50% as topdressing during the tuber expansion period of ginger). Nitrogen fertilizer was applied as urea (46% N) at 400 kg ha^-1^, phosphorus fertilizer as P_2_O_5_ at 200 kg ha^-1^, potassium fertilizer as K_2_O at 600 kg ha^-1^, and cattle manure organic fertilizer at 6000 kg ha^-1^. The amount of fertilizer was based on the recommended amount of local production, and the remaining field management measures were consistent with the local high-yield fields.

**Table 2:** Crop planting density of ginger/maize intercropping system.

Crop	Treatment	Row width	Planting density plants ha^-1^
Ginger		T1	55000
		T2	55000
		T3	61800
	SG		62500
Maize		T1	42500
		T2	28300
		T3	21250
	SM		40000

T1 represents that the planting row width between ginger and maize is 2 m. T2 represents that the planting row width between ginger and maize is 3 m. T3 represents that the planting row width between ginger and maize is 4 m. SM represents that the sole cropping of maize, and SG represents that the sole cropping of ginger

### 2.4 Competition indices and monetary advantages

#### 2.4.1 Yield and system productivity index

The grain yield of each crop was obtained from an experimental plot measuring 48 m^2^ (12 × 4 m). Maize yield was converted to approximately 14% water content after air-drying, and ginger yield was measured as fresh weight. Finally, the System Productivity Index (SPI) was determined using the following equation (Odo, 1991):


$$SPI=\left(\frac{S_{A}}{L_{B}}\times L_{b}\right)+S_{a}$$

where S_A_ indicates the yield of sole-cropped maize, L_B_ indicates the yield of sole-cropped ginger (t ha^-1^); Sa denotes the yield of intercropped maize, and Lb denotes the yield of intercropped ginger (t ha^-1^).

#### 2.4.2 Land Equivalent Ratio

Land Equivalent Ratio (LER) refers to the ratio of income when two or more crops are intercropped on the same farmland to the income of each crop when monocropped. Here, income typically represents crop yield. It is an important indicator for measuring the degree of yield increase in intercropping compared with monocropping, and land-use efficiency. The formula is as follows:


LERt=(LERmaize+LERginger)

$$LERm=\frac{Yim}{Ysm}$$

$$LERg=\frac{Yig}{Ysg}$$

where, LER_t_, LER_m_ and LER_g_ are the total combined and partial LER of maize and ginger, respectively. Y_im_, Y_sm_, Y_ig_, and Y_sg_ indicate the yields of intercropped maize, and Y_sm_ indicates the yield of sole-cropped maize (t ha^-1^); Y_ig_ represents the yield of intercropped ginger, and Y_sg_ represents the yield of sole-cropped ginger (t ha^-1^), respectively.

#### 2.4.3 Relative crowding coefficient

This index is used to measure the degree of crowding among individuals or groups of crops in intercropping system, reflecting the intensity of resource competition—light, water, nutrients, and space—among different crops and the same types of crops. The formula is as follows:


$$Km=\frac{Yim\times Fg}{\left(Ysm-Yim\right)\times Fm}$$

$$Kg=\frac{Yig\times Fm}{\left(Ysg-Yig\right)\times Fg}$$

where Y_im_ and Y_ig_ represent the yields of intercropped maize and ginger respectively, and Y_sm_ and Y_sg_ represent the yields of sole-cropped maize and ginger (t ha^-1^). F_m_ and F_g_ represent the sowing proportions of land for maize and ginger, respectively, during intercropping.

#### 2.4.4 Aggressivity

Degree of advantage of one crop in intercropping system over another crop competing for resources. The formula is as follows:


$$Am=\frac{Ymg}{YmZm}-\frac{Ygm}{YgZg}$$

$$Ag=\frac{Ygm}{YgZg}-\frac{Ymg}{YmZm}$$

where Z_m_ represents the sowing proportion of the land used for maize and Z_g_ represents the sowing proportion of the land used for ginger. If the A value is 0, the two crops have the same competitiveness; if A_m_ is positive, maize has a competitive advantage; and if A_g_ is positive, ginger has a competitive advantage.

This index quantitatively describes the competitive relationship among different crops in intercropping system, reflecting the degree of competitive influence of one crop on another in terms of growth, yield and other aspects (Lithourgidis et al., 2011). The calculation formula is as follows:


$$CRmg=\frac{LERmaize}{LERginger}\times \frac{Zg}{Zm}$$

$$CRgm=\frac{LERginger}{LERmaize}\times \frac{Zm}{Zg}$$

where CR_mg_ is > 1, maize has a stronger competitive ability than ginger. When CR_mg_ is< 1, maize has a weaker competitive ability than ginger.

#### 2.4.5 Actual yield loss

This is an indicator of the yield loss in intercropping system compared with monocropping. The formula is as follows:


AYLmaize={[YimZimYmZmm]−1}

AYLginger={[YigZigYgZgg]−1}

where Z_im_ represents the sowing proportion of maize in intercropping system, Z_ig_ represents the sowing proportion of ginger in intercropping system, Z_mm_ represents the sowing proportion of sole-cropped maize, and Z_gg_ represents the sowing proportion of sole-cropped ginger. Compared to monocropping, AYL_maize_ and AYL_ginger_ describe the relative decrease in the yield of maize and ginger per sowing proportion in ginger/maize intercropping system, respectively. An overall positive AYL represents the advantage of intercropping system, whereas a negative AYL represents a disadvantage.

#### 2.4.6 Intercropping advantage

The calculation formula is as follows:


IAmaize=AYLmaize×Pmaize

IAginger=AYLginger×Pginger

where Pmaize and Pginger represent the commercial values of maize (in 2017: 0.45 USD per kg; in 2018: 0.44 USD per kg) and ginger (in 2017: 0.98 USD per kg; in 2018: 1.02 USD per kg), respectively. The average price per kilogram was obtained from a local grain market during the planting season. Overall, a higher IA value indicates a more profitable cropping system.

#### 2.4.7 Monetary advantage index

The calculation formula is as follows:


$$MAI=\frac{value\;of\;combined\;intercrops}{LER}\times \left(LER-1\right)$$

The output value of the intercropping system is the sum of the yield of the two crops multiplied by the market price. The higher the MAI value, the greater the profit of the planting system (Ghosh, 2004).


$$BCR=\frac{Total\;revenue}{Total\;cost}$$

The total input includes the cost of seeds, fertilizers (nitrogen, phosphorus, and potassium), pesticides, and labor. Ginger and maize prices are based on market prices during the Chongqing season, and the labor cost is 8.90 USD per person per day. During the field management period, 3 laborers per hectare were employed. For sowing and harvesting operations, the labor input was 30 laborers per hectare. The local market prices of ginger and maize in the first year (2017) and second year (2018) by the measured yields of these two crops each year were used to calculate gross income (GI). Additionally, a benefit-cost ratio (BCR) analysis was conducted to evaluate the economic feasibility of intercropping system (Yang et al., 2018). BCR > 1 indicates that intercropping system has good economic benefits; a BCR less than 1 may lead to losses.

### 2.5 Statistical analysis

The experimental data were analyzed by one-way analysis of variance (ANOVA) and multivariate analysis of variance (MANOVA) using IBM SPSS Statistics 25.0 software (IBM Corporation, Armonk, New York, USA). ANOVA was performed using Statistix 8.1 (Analytical Software, Tallahassee, Florida, USA). At a significance level of p ≤ 0.05, the least significant difference (LSD) of the Student-Newman-Keuls test was used for *post-hoc* comparison for separation and testing.

## 3 Results

### 3.1 Yield performance

Maize plant type and row width had significant effects on ginger yield, maize yield and total yield in ginger/maize intercropping system, and the interaction of plant type × row width × year also had significant effects on yield, among which row width was the main factor affecting the yield of intercropping system (**Table 3**). For ginger/maize intercropping system, the yields of ginger, maize, and total yield under the ZH 311 treatment were significantly higher than those under the ZH 2 treatment. When intercropped with ZH 311, compared with the non-shaded SG treatment, the ginger yield increased by 44.54%, 14.53%, and 8.65% in 2017 and by 52.17%, 26.52%, and 18.90% in 2018 under T1, T2, and T3 treatments, respectively. When intercropped with ZH 2, compared with the non-shaded SG treatment, the ginger yield increased by 31.09%, 12.78%, and 2.85% in 2017 and by 46.71%, 22.37%, and 9.90% in 2018 under T1, T2, and T3 treatments, respectively. With an increase in row width, the yields of ginger and maize and the total yield in the ginger/maize intercropping system decreased Significantly. Compared with T1, the ginger yield in T2 decreased by 17.13%, and compared with T2, the ginger yield in T3 decreased by 7.51%. Compared with T1, the maize yield in T2 decreased by 24.47%, and compared with T2, the maize yield in T3 decreased by 17.22%. Compared with T1, the total yield in T2 decreased by 17.80%, and compared with T2, the total yield in T3 decreased by 8.32%. Except for the two data of maize intercropping ZH 311 at 3 m row width and maize intercropping ZH 2 at 2 m row width, the ginger yield, maize yield and total system yield in 2017 were always higher than those in 2018 under the same plant type and row width treatment. It shows that the yield of intercropping system changes with climatic conditions, but the yield-increasing effect of dominant configuration is relatively stable as a whole. The SPI of all intercropping treatments was greater than zero, which confirmed that the ginger/maize intercropping system had an absolute advantage in productivity relative to the monoculture. The SPI of flat type maize (ZH 311) was significantly higher than that of compact type (ZH 2), indicating that the former was more conducive to improving the overall productivity of the system. With the increase of row width from 2m (T1) to 4m (T3), SPI showed a significant downward trend, and T1 treatment SPI was the highest (66.05), which was significantly better than T2 and T3. In order to eliminate the interference of different planting row spacing on yield comparison and realize the scientific comparison of crop yield on the same basis, the original yield data were corrected and analyzed by calculating the yield per plant (**Table 4**). From the perspective of yield per plant, the yield per plant of intercropping maize under T3 treatment was significantly higher than that under T1 treatment, indicating that T3 treatment was more conducive to improving the resource acquisition ability and material accumulation level of maize per plant. The yield per plant of intercropping ginger under T1 treatment was significantly higher than that under T3 treatment, which reflected the interspecific promotion effect of maize on ginger growth under T1 treatment. The comparison of total yield per plant in ginger/maize intercropping system showed that the promotion effect of flat maize ZH 311 on intercropping ginger yield was higher than that of compact maize ZH 2.

**Table 3:** Crop yield and system productivity index (SPI) of ginger/maize intercropping system.

Variety	Row width	Grain yield (t ha^-1^)						SPI	
		Ginger		Maize		Total			
		2017	2018	2017	2018	2017	2018	2017	2018
ZH 311	T1	49.65±0.61a	47.34±0.42a	5.20±0.03b	4.70±0.07b	54.84±0.63a	52.04±0.36a	69.83±1.16a	65.08±0.42a
	T2	39.34±0.56b	39.36±0.94b	3.80±0.03c	3.53±0.02c	43.14±0.57b	42.89±0.96b	54.09±0.96b	52.66±0.56b
	T3	37.32±0.12c	36.99±0.71c	3.25±0.01d	2.87±0.03d	40.57±0.12c	39.86±0.73c	49.94±0.33b	47.80±0.43c
	Average	42.10	41.23	4.08	3.70	46.18	44.93	—	—
CK	SM	—	—	8.86±0.12a	8.26±0.08a	8.86±0.12e	8.26±0.08e	—	—
	SG	34.35±0.40d	31.11±0.76d	—	—	34.35±0.40d	31.11±0.78d	—	—
ZH 2	T1	45.03±0.78a	45.64±0.61a	4.53±0.06b	4.33±0.06b	49.56±0.82a	49.96±0.58a	65.09±1.18a	64.19±0.28a
	T2	38.74±1.12b	38.07±1.11b	3.54±0.03c	3.30±0.03c	42.28±1.14b	41.37±1.13b	53.42±1.39b	52.23±1.44b
	T3	35.33±1.01c	34.19±0.40c	2.92±0.04d	2.69±0.03d	38.25±0.99c	36.88±0.38c	48.27±1.20c	45.73±0.08c
	Average	39.70	39.30	3.66	3.44	43.36	42.74	—	—
CK	SM	—	—	7.76±0.12a	7.25±0.06a	7.76±0.12e	7.25±0.06e	—	—
	SG	34.35±0.40c	31.11±0.78d	—	—	34.35±0.40d	31.11±0.78d	—	—
F value	Variety (V)	20.08**		295.79**		34.24**		—	—
	Row (R)	3093.34**		11717.22**		2510.71**		—	—
	Years (Y)	12.61**		149.85**		20.44**		—	—
	V×R×Y	3.13**		22.10**		2.89**		—	—

The letters in the same column indicated that there was a significant difference between maize and ginger treatments at the p ≤ 0.05. * is significant at the 0.05 level, ** is significant at the 0.01 level, and ns is not significant, The data annotation value is mean±standard error (Mean±SE). ZH 311 is flat type maize and ZH 2 is compact type maize. T1 represents that the planting row width between ginger and maize is 2 m. T2 represents that the planting row width between ginger and maize is 3 m. T3 represents that the planting row width between ginger and maize is 4 m. SM, Sole cropping of maize; SG, Sole cropping of ginger; The average yield of different row-width treatments in the intercropping system.

**Table 4:** Crop yield per plant under different treatments in ginger/maize intercropping system.

Variety	Row width	Grain yield (kg plant^-1^)					
		Ginger		Maize		Total	
		2017	2018	2017	2018	2017	2018
ZH 311	T1	0.90±0.01a	0.86±0.01a	0.12±0.00d	0.11±0.00d	1.03±0.01a	0.97±0.01a
	T2	0.72±0.01b	0.72±0.02b	0.13±0.00c	0.13±0.00c	0.85±0.01b	0.84±0.02b
	T3	0.60±0.00c	0.60±0.01c	0.15±0.00b	0.14±0.00b	0.75±0.00c	0.73±0.01c
CK	SM	—	—	0.22±0.00a	0.21±0.00a	0.22±0.00e	0.21±0.00e
	SG	0.55±0.01d	0.50±0.01d	—	—	0.55±0.01d	0.50±0.01d
ZH 2	T1	0.82±0.02a	0.83±0.01a	0.11±0.00d	0.10±0.00d	0.93±0.01a	0.93±0.01a
	T2	0.70±0.02b	0.69±0.02b	0.13±0.00c	0.12±0.00c	0.83±0.02b	0.81±0.02b
	T3	0.57±0.02c	0.55±0.01c	0.14±0.00b	0.13±0.00b	0.71±0.01c	0.67±0.01c
CK	SM	—	—	0.19±0.10a	0.18±0.00a	0.19±0.00e	0.18±0.00e
	SG	0.55±0.01c	0.50±0.01c	—	—	0.55±0.01d	0.50±0.01d

The letters in the same column indicated that there was a significant difference between maize and ginger treatments at the p ≤ 0.05. * is significant at the 0.05 level, ** is significant at the 0.01 level, and ns is not significant, The data annotation value is mean±standard error (Mean±SE). ZH 311 is flat type maize and ZH 2 is compact type maize. T1 represents that the planting row width between ginger and maize is 2 m. T2 represents that the planting row width between ginger and maize is 3 m. T3 represents that the planting row width between ginger and maize is 4 m. SM, Sole cropping of maize; SG, Sole cropping of ginger.

### 3.2 Resource use efficiency

**Figure 3:**
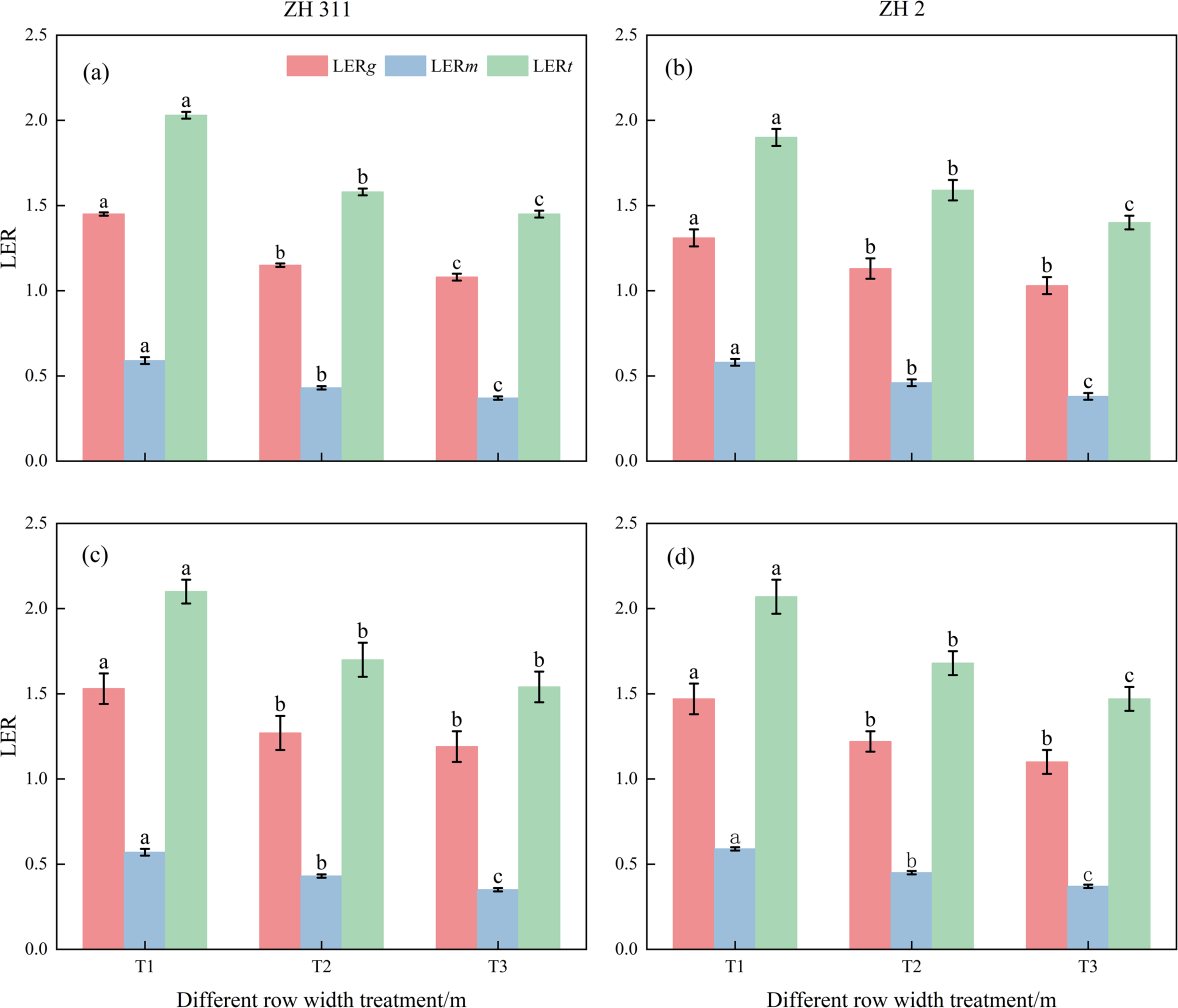
LER for ginger (LERg) and maize (LERm) for three set of intercropping systems from 2017-2018, where LERt represents the total LER of the system. **(a)** LER in the intercropping of ginger and flat maize ZH 311 in 2017. **(b)** LER in the intercropping of ginger and compact maize ZH 2 in 2017. **(c)** LER in the intercropping of ginger and flat maize ZH 311 in 2018. **(d)** LER in the intercropping of ginger and compact maize ZH 2 in 2018. Different letters in the same LER (bars of the same color in the chart) indicate statistically significant differences at the p ≤ 0.05. T1 means that the planting row width is 2 m, T2 means that the planting row width is 3 m, T3 means that the planting row width is 4 m.

In the ginger/maize intercropping system, the LER_g_ under all treatments was greater than 1, while the LER_m_ under all treatments was less than 1 (**Figure 3**). This indicates that ginger in the ginger/maize intercropping system had higher land use efficiency and yield advantage, whereas maize showed the opposite trend. Additionally, the LER_t_ of the intercropping system reached a maximum of 2.10 (**Figure 3a**). In the ginger/maize intercropping system, significant differences were observed in the LER_m_ among different row width treatments. When ginger was intercropped with the flat-type maize ZH 311, the LER_m_ of maize under all treatments was lower than that when ginger was intercropped with the compact-type maize ZH 2. The LER_g_ under the T1 treatment was significantly higher than that under the T2 and T3 treatments. Except for 2017, when ginger was intercropped with ZH 311, there was no significant difference in LER_g_ of ginger between the T2 and T3 treatments. The LER_t_ of the intercropping system under the T1 treatment was also significantly higher than that under the T2 and T3 treatments. The LER_t_ values of T2 and T3 treatments were 1.64 and 1.47, respectively, which were significantly decreased by 26.08% and 38.10% compared with the T1 treatment. With the increase in row width, LER_g_, LER_m_, and LER_t_ all showed a decreasing trend, resulting in a significant reduction in land use efficiency and yield advantage. Therefore, under the T1 treatment where ginger is intercropped with the flat-type maize ZH 311, the ginger/maize intercropping system achieves the highest yield advantage and land use efficiency.

### 3.3 Competition dynamics

Across all treatments over two years, the growth advantage coefficient of maize (K_maize_) was consistently higher than that of ginger (K_ginger_), indicating that maize had a stronger growth advantage in the intercropping system (**Table 5**). Specifically, when ginger was intercropped with the compact-type maize ZH 2, the K_maize_ under the T1 treatment reached the highest value of 2.96. With the increase in row width, K_ginger_ decreased significantly, while K_maize_ exhibited a trend of first decreasing and then increasing, following the order T1 > T3 > T2. In 2017, when ginger was intercropped with ZH 2, the yield of intercropped ginger under the T3 treatment was slightly lower than that of sole-cropped ginger (SG); thus, K_ginger_ showed a positive value here. In all other treatments, however, the yield of intercropped ginger in the intercropping system was significantly higher than that of sole-cropped ginger, resulting in a negative Kginger. In the ginger/maize intercropping system, the growth advantage coefficients always satisfied K_maize_ > K_ginger_. This indicates that during the ginger-maize coexistence period, maize had greater growth advantage and competitiveness compared to ginger. Furthermore, compared with intercropping with ZH 311, the growth disadvantage of ginger was more pronounced when intercropped with ZH 2.

**Table 5:** Values of relative crowding coefficient (K), aggressivity (A), and competition ratio (CR) for ginger and maize in different intercropping systems.

Variety	Row width	Relative crowding coefficient				Aggressivity				Competitive ratio			
		K_ginger_		K_maize_		A*_gm_*		A*_mg_*		CR*_gm_*		CR*_mg_*	
		2017	2018	2017	2018	2017	2018	2017	2018	2017	2018	2017	2018
ZH 311	T1	-1.62±0.01a	-1.47±0.09ns	2.85±0.11a	2.65±0.13a	0.40±0.03a	0.58±0.12a	-0.40±0.03c	-0.58±0.11c	1.23±0.02a	1.34±0.08a	0.81±0.01c	0.75±0.04c
	T2	-2.63±0.07b	-1.70±0.27ns	2.26±0.06b	2.24±0.02b	-0.19±0.03b	-0.02±0.09b	0.19±0.03b	0.02±0.09b	0.89±0.01b	0.99±0.06b	1.12±0.01b	1.02±0.05b
	T3	-2.92±0.43b	-1.57±0.35ns	2.62±0.07a	2.39±0.02b	-0.70±0.05c	-0.45±0.07c	0.70±0.05a	0.45±0.07a	0.66±0.02c	0.77±0.03c	1.53±0.04a	1.33±0.06a
ZH 2	T1	-2.13±0.14ns	-1.59±0.11ns	2.82±0.18ns	2.96±0.09a	0.21±0.07a	0.41±0.07a	-0.21±0.07c	-0.41±0.07c	1.12±0.04a	1.23±0.04a	0.89±0.03c	0.81±0.03c
	T2	-3.42±0.91ns	-1.89±0.25ns	2.52±0.11ns	2.51±0.07b	-0.32±0.07b	-0.19±0.02b	0.32±0.07b	0.19±0.02b	0.82±0.03b	0.90±0.01b	1.22±0.05b	1.13±0.01b
	T3	2.44±10.76ns	-3.88±1.35ns	2.72±0.11ns	2.66±0.06b	-0.81±0.07c	-0.71±0.06c	0.81±0.07a	0.71±0.06a	0.61±0.03c	0.66±0.03c	1.65±0.07a	1.52±0.06a

After the same column of data, different lowercase letters (eg: a、b、c) indicated that the difference between different intercropping treatments was statistically significant at the P ≤ 0.05 level, and ns is not significant. The data annotation value is mean±standard error (Mean±SE). ZH 311 is flat type maize and ZH 2 is compact type maize. T1 means that the planting row width is 2 m, T2 means that the planting row width is 3 m, T3 means that the planting row width is 4 m.

In terms of competitive relationships, under the T1 treatment, the aggressiveness of ginger towards maize (A_gm_) > 0 and the competitive ratio of ginger to maize (CR_gm_) > 1, indicating that ginger became the dominant species under this condition. Whereas in the T2 and T3 treatments, the aggressiveness of maize towards ginger (A_mg_) > 0 and the competitive ratio of maize to ginger (CR_mg_) > 1, with maize becoming the dominant species instead. Furthermore, in the ginger/maize intercropping system, A_gm_ and CR_gm_ under the ZH 311 (flat-type maize) treatment were significantly higher than those under the ZH 2 (compact-type maize) treatment, while Amg and CR_mg_ were significantly lower than those under the ZH 2 treatment. This demonstrates that ginger exhibited stronger resource competitiveness when intercropped with the flat-type maize ZH 311. With the increase in row width, Agm and CR_gm_ decreased significantly, whereas A_mg_ and CR_mg_ increased significantly—this further reflects the regulatory effect of row width configuration on interspecific competitive dynamics.

### 3.4 Economic performance

Across all treatments over two years, the values of AYL_ginger_, AYL_maize_, and AYL_total_ were all greater than 0, revealing the yield advantage of the ginger/maize intercropping system compared with sole-cropped ginger and maize (**Table 6**). Under the ZH 311 (flat-type maize) treatment, AYL_ginger_ of the intercropping system was generally higher than that under the ZH 2 (compact-type maize) treatment, while AYL_maize_ was generally lower than that under the ZH 2 treatment. Under the T1 and T3 treatments, AYL_total_ when intercropped with ZH 311 decreased compared with that when intercropped with ZH 2, whereas it increased under the T2 treatment. With the increase in row width, AYL_ginger_ and AYL_total_ showed a decreasing trend, while AYL_maize_ showed an increasing trend. Under the T1 and T3 treatments, AYL_ginger_ reached the maximum value of 1.23 and the minimum value of 0.30, respectively (two-year average, the same below). Under the T1 treatment, when ginger was intercropped with ZH 311, the ginger/maize intercropping system had the highest AYL_total_, reaching 1.96.

**Table 6:** Actual yield losses (AYL), intercropping advantage (IA), and monetary advantage indices (MAI) of ginger/maize intercropping under different intercropping systems.

Variety	Row width	Actual yield loss						Intercropping advantage						MAI	
		AYLginger		AYLmaize		AYLtotal		IAginger		IAmaize		IAtotal			
		2017	2018	2017	2018	2017	2018	2017	2018	2017	2018	2017	2018	2017	2018
ZH 311	T1	1.17±0.00a	1.29±0.08a	0.76±0.03b	0.71±0.04b	1.93±0.03a	1.99±0.04a	7.60±0.03a	9.01±0.55a	2.29±0.08b	2.12±0.12b	9.88±0.09a	11.13±0.44a	181.15±2.97a	180.41±4.53a
	T2	0.53±0.01b	0.69±0.08b	0.72±0.03b	0.71±0.01b	1.24±0.03c	1.40±0.08b	3.43±0.04b	4.84±0.58b	2.15±0.08b	2.13±0.03b	5.57±0.11b	6.97±0.56b	102.34±2.48b	117.27±8.65b
	T3	0.33±0.02c	0.46±0.06b	1.02±0.03a	0.91±0.01a	1.35±0.02b	1.37±0.06b	2.13±0.11c	3.20±0.46b	3.06±0.10a	2.73±0.03a	5.20±0.01c	5.93±0.45b	82.35±1.08c	93.53±7.70b
ZH 2	T1	0.97±0.04a	1.21±0.08a	0.75±0.04b	0.79±0.02b	1.72±0.05a	2.00±0.09a	6.29±0.28a	8.43±0.57a	2.26±0.14b	2.37±0.07b	8.54±0.27a	10.80±0.60a	144.67±4.89a	171.41±6.37a
	T2	0.51±0.05b	0.64±0.04b	0.82±0.04b	0.82±0.02b	1.33±0.06b	1.45±0.07b	3.28±0.31b	4.43±0.30b	2.47±0.16b	2.46±0.08b	5.75±0.33b	6.89±0.38b	96.85±6.43b	111.79±6.22b
	T3	0.26±0.03c	0.33±0.05c	1.07±0.05a	1.04±0.03a	1.33±0.05b	1.37±0.07b	1.67±0.20c	2.33±0.37c	3.21±0.13a	3.12±0.10a	4.88±0.21b	5.45±0.39b	68.78±4.78c	78.45±5.49c

After the same column of data, different lowercase letters (eg: a、b、c) indicated that the difference between different intercropping treatments was statistically significant at the P ≤ 0.05 level. The data annotation value is mean±standard error (Mean±SE). ZH 311 is flat type maize and ZH 2 is compact type maize. T1 means that the planting row width is 2 m, T2 means that the planting row width is 3 m, T3 means that the planting row width is 4 m.

Across all treatments over two years, both IAginger and IAmaize were positive, indicating that the ginger/maize intercropping system had a significant yield advantage over sole cropping. In the ginger/maize intercropping system, IAginger, IAtotal, and MAI under the ZH 311 treatment were higher than those under the ZH 2 treatment, while IAmaize showed the opposite trend. With the increase in row width, IAginger, IAtotal, and MAI decreased significantly, whereas AYLmaize increased significantly. Under the T1 treatment, when ginger was intercropped with ZH 311, the observed values of IAtotal and MAI were the highest (10.51 and 180.78, respectively). Under the T3 treatment, when ginger was intercropped with ZH 2, the observed values of IAtotal and MAI were the lowest (5.17 and 72.62, respectively). This confirms that the ginger/maize intercropping system achieved the highest economic benefits when ginger was intercropped with ZH 311 under a 2 m row width (T1 treatment).

The results of gross income (GI), net income (NI), and benefit-cost ratio (BCR) for the ginger/maize intercropping system are presented (**Table 7**). Among these, the BCR of the ginger/maize intercropping system was 1.28 to 1.65 times that of sole-cropped maize (SM). When ginger was intercropped with ZH 311 (flat-type maize), the ginger/maize intercropping system achieved the highest GI under the T1 treatment, with values of 51,200 USD ha^-1^ (2017) and 50,300 USD ha^-1^ (2018), respectively. In contrast, when ginger was intercropped with ZH 2 (compact-type maize), the system had the lowest GI under the T3 treatment, at 36,100 USD ha^-1^ (2017) and 25,700 USD ha^-1^ (2018), respectively. Among the 8 treatments across 5 planting patterns, the planting systems ranked in descending order of NI as follows: T1 > T2 > T3 > sole-cropped ginger (SG) > sole-cropped ginger (SM). Under the ZH 311 treatment, GI, NI, and BCR of the ginger/maize intercropping system were all higher than those under the ZH 2 treatment. Additionally, with the increase in row width, GI, NI, and BCR all decreased significantly. In the ginger/maize intercropping system: When intercropped with ZH 311, the GI of the T1 treatment (two-year average, the same below) was 55.06% higher than that of SG, and 12.30 times higher than that of SM; When intercropped with ZH 2, the GI of the T1 treatment was 44.83% higher than that of SG, and 13.16 times higher than that of SM. Analysis of the BCR showed that the intercropping systems ranked in descending order of BCR as follows: T1 > T2 > SG > T3 > SM.

**Table 7:** Economic analysis of ginger/maize intercropping under different intercropping systems from 2017 to 2018 according to the value of the currency (USD) in respective years.

Variety	Row width	Gross income (×10^4^ USD ha^-1^)		Cost (×10^4^ USD ha^-1^)		Net income (×10^4^ USD ha^-1^)		Benefit-cost ratio	
		2017	2018	2017	2018	2017	2018	2017	2018
ZH 311	T1	5.12±0.06a	5.03±0.04a	1.92±0.01b	1.83±0.01b	3.20±0.06a	3.20±0.04a	2.67±0.03a	2.75±0.02a
	T2	4.04±0.05b	4.17±0.10b	1.91±0.01b	1.83±0.01b	2.13±0.05b	2.34±0.10b	2.11±0.03b	2.28±0.06b
	T3	3.82±0.01c	3.89±0.07c	1.99±0.01a	1.86±0.01a	1.82±0.01c	2.03±0.08c	1.91±0.01d	2.04±0.05c
CK	SM	0.40±0.01e	0.36±0.01e	0.24±0.01d	0.23±0.01d	0.17±0.01e	0.14±0.01e	1.69±0.03e	1.60±0.01e
	SG	3.38±0.04d	3.17±0.08d	1.69±0.01c	1.62±0.01c	1.69±0.04d	1.55 ± .0.08d	2.00±0.02c	1.95±0.05d
ZH 2	T1	4.63±0.08a	4.84±0.06a	1.92±0.01b	1.84±0.01b	2.71±0.08a	3.00±0.06a	2.41±0.04a	2.63±0.03a
	T2	3.97±0.11b	4.03±0.11b	1.92±0.01b	1.84±0.01b	2.05±0.11b	2.18±0.11b	2.07±0.06b	2.18±0.06b
	T3	3.61±0.10c	3.57±0.05c	2.00±0.01a	1.92±0.01a	1.60±0.10c	1.65±0.05c	1.80±0.05c	1.86±0.03c
CK	SM	0.35±0.01d	0.32±0.01e	0.24±0.01d	0.23±0.01d	0.11±0.01d	0.09±0.01d	1.47±0.02d	1.38±0.01d
	SG	3.38±0.04c	3.17±0.08d	1.69±0.01c	1.62±0.01c	1.69±0.04c	1.55±0.08c	2.00±0.03b	1.96±0.05c

After the same column of data, different lowercase letters (eg: a、b、c) indicated that the difference between different intercropping treatments was statistically significant at the P ≤ 0.05 level. The data annotation value is mean±standard error (Mean±SE). ZH 311 is flat type maize and ZH 2 is compact type maize. T1 means that the planting row width is 2 m, T2 means that the planting row width is 3 m, T3 means that the planting row width is 4 m.The commercial values of the crops were set as follows: maize (in 2017: 0.45 USD per kg; in 2018: 0.44 USD per kg) and ginger (in 2017: 0.98 USD per kg; in 2018: 1.02 USD per kg).

## 4 Discussion

In intercropping systems, different combinations of crop plant types and row width collectively influence system productivity by regulating the crop growth environment and interspecific competition. Plant type modulates crop biomass production by affecting canopy light interception and ventilation conditions (Bo et al., 2019), while row width influences land and light use efficiency in the intercropping system by altering the spatial distribution of crops (Wu et al., 2023). The results of this study indicate that the ginger-maize intercropping system can achieve synergistic improvement in yield and economic benefits by optimizing maize plant type and row width configurations, while significantly enhancing the system’s land equivalent ratio, system productivity index, and benefit-cost ratio. Compared with monocropping, the yield of maize was significantly reduced in the ginger-maize intercropping system, while the yield of ginger and the total system yield were greatly increased, showing a typical yield-increasing effect of interspecific complementarity in intercropping. This phenomenon is in stark contrast to the report that crop yield in maize/legume intercropping system is significantly lower than that in sole-cropped (Raza et al., 2023). This difference primarily stems from the intrinsic distinctions in light ecological requirements: soybean, as a light-demanding crop, experiences limited photosynthetic efficiency under maize shading, whereas the shade-tolerant ginger fully utilizes the shaded environment provided by maize to promote growth, while its per-plant yield advantage ensures maize’s own output (Luo et al., 2024). Consistently, wheat/faba bean intercropping also achieves the improvement of system productivity by optimizing management measures and relying on the growth complementarity between crops, which further confirms the importance of reasonable crop matching (Gidey et al., 2025; Xiao et al., 2018). Similar yield-increasing mechanisms were observed in the intercropping system of pear orchards (Wang and Gu, 2016). We also found that under the condition of high temperature and heat in summer, intercropping with kiwifruit and citrus can create a good growth environment for ginger, improve photosynthetic performance, and achieve a significant increase in yield (Peng et al., 2024), indicating that reasonable crop matching and niche complementarity are the key to improving intercropping advantages.This study found that the flat-type maize ZH 311 was significantly better than the compact maize ZH 2 in improving the productivity of the ginger/maize intercropping system. The reason was that the flat canopy could provide a more suitable shade microenvironment for ginger, which was contrary to the research results that the compact maize had more intercropping advantages in the sweet potato/maize intercropping system (Wang, 2015). Moreover, the yield reduction caused by increased row width confirmed the inhibitory effect of reduced shading intensity on ginger growth, which stands in complete contrast to the yield promotion observed in maize/soybean intercropping systems with increased row width (Li, 2024). This indicates that the optimal allocation of intercropping systems must fully consider the ecological characteristics of the main crop and establish a cultivation model based on complementary growth needs.

The economic viability of intercropping systems is jointly determined by resource use efficiency and market value. This study demonstrates that rational planting design can significantly enhance the economic benefits of intercropping systems. The ginger/maize intercropping system achieved a benefit-cost ratio 1.28–1.65 times higher than that of maize monoculture, surpassing traditional intercropping patterns such as maize/peanut (1.21–1.25 times) (Lu, 2018), maize/soybean (1.33–1.47 times) (Li et al., 2023), and maize/sweet potato (0.90–0.93 times) (Zhao et al., 2018). This superior performance is primarily attributed to the dual effects of ginger’s market premium as a high-value cash crop and its yield enhancement. The comprehensive advantages observed under the flat-type maize ZH 311 treatment—in terms of land equivalent ratio, gross income, net income, benefit-cost ratio, and the per-plant yields—confirm the regulatory role of plant type selection on economic returns. Conversely, the decline in economic indicators with increased row width underscores the necessity of maintaining moderate shading to ensure ginger’s economic output. Compared with the maize/soybean intercropping system, ginger intercropping achieved more significant economic gains through higher product value, which coincides with the strategy of improving the profitability of the system through the combination of high value-added crops in the cotton/soybean intercropping system (Lv et al., 2025). This confirms that the economic advantages of intercropping systems stem not only from yield increases but also from the synergistic effects of crop combination market value and resource use efficiency. Analysis of interspecific competition dynamics provides an explanation for the observed phenomena. Under the flat-type maize treatment, the K was lower, while A and CR were higher, indicating that the intercropping system established a more balanced interspecific relationship by weakening the competitive advantage of maize and improving the competitiveness of ginger.With the increase of row width, the canopy ventilation and light transmission conditions were improved, which intensified the intensity of interspecific resource competition. The shade-tolerant superiority of ginger was weakened and its competitive capacity gradually declined, whereas the competitive advantage of maize was further strengthened. This finding is inconsistent with previous studies, which reported that the competitiveness of soybean was significantly lower under flat-type maize than under compact-type maize (Wu, 2007). This crop-specific response in competition dynamics again highlights the advantage of ginger/maize intercropping design based on niche complementarity: soybean, as a light-demanding crop, exhibits enhanced competitiveness in wider row width, whereas ginger, as a shade-tolerant crop, demonstrates competitive advantages under moderate shading conditions (Yong et al., 2015). The differentiation in resource utilization habits of different crops is the core cause of the variation in interspecific competition intensity and economic benefits in intercropping systems. In agricultural production, we can improve land use efficiency and increase production and income by designing similar niche complementary intercropping planting systems.

## 5 Conclusions

This study shows that maize plant type and intercropping row width can affect the yield, interspecific competition and economy of ginger/maize intercropping system by regulating canopy microenvironment and interspecific niche complementarity. This result echoes the research hypothesis. The canopy of flat-spreading maize ZH 311 can form moderate shading, alleviate high light and high temperature stress, strengthen the niche complementary effect with ginger, reduce interspecific competition, and take into account its own photosynthetic efficiency and ginger growth needs; under the 2 m row spacing treatment, the interspecific competition can be further weakened, so that ginger can occupy a competitive advantage, and the two cooperate to achieve the simultaneous improvement of land use efficiency and crop productivity. In summary, in the ginger/maize intercropping system, the use of 2 m row spacing configuration and the priority selection of flat maize ZH 311 is the preferred technical path for increasing yield and efficiency in the ginger/maize intercropping system, and provides a mechanism-level reference for the optimization of similar intercropping patterns.

The ginger/maize intercropping model proposed in this study is a typical model for efficient utilization of intensive agricultural land, which has important reference value for promoting green and sustainable development of agriculture. It is suitable for areas with tight cultivated land and similar light and heat conditions (such as warm temperate semi-humid areas), which can help solve the problem of improving the quality and efficiency of small-scale farmers ‘ production. The core idea of intercropping configuration optimization revealed in this study has cross-system universality, which can be extended to various grain and economic intercropping systems such as maize/soybean and wheat/faba bean, and provide field practice basis for the play of interspecific complementary effects under different crop intercropping modes. In the future, we can focus on the adaptability of the model in different ecological zones and the optimization of planting parameters, improve its sustainability and large-scale promotion value, and provide technical support for high-quality agricultural development, food and agricultural ecological security.

## Data Availability

The original contributions presented in the study are included in the article/supplementary material, further inquiries can be directed to the corresponding author/s.
